# Long-term outcomes of endoscopic resection and tailored adjuvant radiotherapy for sinonasal intestinal-type adenocarcinoma: a historical single-center cohort study in 200 patients

**DOI:** 10.3389/fonc.2025.1522113

**Published:** 2025-03-28

**Authors:** Quentin-Alexandre Parys, Matthias De Witte, Esther Hauben, Paul M. Clement, Robert Hermans, Thomas Decramer, Johannes van Loon, Sandra Nuyts, Mark Jorissen, Vincent Vander Poorten, Laura Van Gerven

**Affiliations:** ^1^ Department of Otorhinolaryngology-Head and Neck Surgery, University Hospitals Leuven, Leuven, Belgium; ^2^ Department of Pathology, University Hospitals Leuven, Leuven, Belgium; ^3^ Department of General Medical Oncology, University Hospitals Leuven, Leuven Cancer Institute, Leuven, Belgium; ^4^ Department of Radiology, University Hospitals Leuven, Leuven, Belgium; ^5^ Department of Neurosurgery, University Hospitals Leuven, Leuven, Belgium; ^6^ Research Group Experimental Neurosurgery and Neuroanatomy and Leuven Brain Institute, Catholic University of Leuven, Leuven, Belgium; ^7^ Department of Radiotherapy–Oncology, University Hospitals Leuven, Leuven, Belgium; ^8^ Laboratory of Experimental Radiotherapy, Department of Oncology, Catholic University Leuven, Leuven, Belgium; ^9^ Section of Head and Neck Oncology, Department of Oncology, Catholic University Leuven, Leuven, Belgium; ^10^ Department of Neurosciences, Experimental Otorhinolaryngology, Rhinology Research, Catholic University Leuven, Leuven, Belgium; ^11^ Department of Microbiology, Immunology and Transplantation, Allergy and Clinical Immunology Research Group, Catholic University Leuven, Leuven, Belgium

**Keywords:** sinonasal tumor, intestinal-type adenocarcinoma, endoscopic sinus surgery, skull base, radiotherapy

## Abstract

**Background:**

Sinonasal intestinal-type adenocarcinoma (ITAC) is a rare disease entity. In contrast to most previous studies, this cohort study consists of a substantial number of uniformly treated patients undergoing endoscopic surgery and adjuvant radiotherapy and provides updated insights into survival outcomes and tumor and treatment-related prognostic factors.

**Material and methods:**

We retrospectively analyzed the medical records of 200 patients primarily treated for ITAC between 1992 and 2022 in our tertiary referral center. Descriptive statistics were applied using Kaplan-Meier method. Cox models were used for univariable and multivariable data analysis.

**Results:**

The 5-year overall survival (OS), disease-specific survival (DSS), and local recurrence-free survival (LRFS) rates were 71.4%, 85.1%, and 55.2% respectively. At 10 years, the numbers decreased to 48.2%, 76.2%, and 32.2% respectively. Significant differences were found in OS and DSS between T-groups. Poorly differentiated tumors had decreased DSS compared to well-differentiated tumors (HR: 3.38 [95% CI: 1.20–9.51], p=.0209). Signet-cell differentiation was associated with the poorest survival among poorly differentiated tumors although not reaching significance. In 34.0% of patients, there was local recurrence, with half of the cases detected within the first two years of follow-up but over 10% of recurrence occurring after 10 years. Positive surgical margins (HR: 2.95 [95% CI: 1.29–6.74], p=.0106), local recurrence (HR: 12.28 [95% CI: 5.59–26.99], p<.0001), and distant metastasis (HR: 41.17 [95% CI: 21.58–78.55], p<.0001) negatively affected DSS. Distant metastasis occurred more frequently in poorly differentiated tumors (25.6%) compared to moderately differentiated (9.5%) and well-differentiated tumors (2.5%) (p=.002).

**Conclusions:**

This extensive study focusing on sinonasal ITAC primarily managed through endoscopic resection and radiotherapy, demonstrates that T-classification and tumor differentiation are independent prognostic factors influencing survival. Furthermore, local recurrence, distant metastasis, and positive surgical margins negatively affect OS and DSS.

## 1 Introduction

According to data from the International Agency for Research on Cancer (IARC), malignant tumors affecting the sinonasal cavity are considered rare, compromising only 0.4% of all malignancies worldwide (1). However, in Belgium, these malignancies constitute a larger proportion, comprising 4.6% of newly incident head and neck carcinomas in 2017 (2). Among these, intestinal-type adenocarcinoma (ITAC) emerges as one of the most frequently observed histopathological subtypes. This notable incidence of ITAC is primarily attributed to occupational exposure to hardwood dust (3, 4).

Primary ITAC is histopathologically similar to adenocarcinomas of the intestinal mucosa. Sinonasal ITAC can be further subclassified morphologically and according to histological grade of differentiation into well-differentiated (papillary, tubular, and papillary-tubular type), moderately differentiated (papillary-mucinous and papillary-tubular-mucinous type) and poorly differentiated (mucinous, alveolar goblet cell, and signet-ring type) tumors (4).

Presenting symptoms in patients with ITAC are usually nonspecific, leading to a delay in diagnosis. Diagnosis of sinus tumors is primarily based on endoscopic visualization and biopsy. Cross-sectional imaging through CT and MRI is recommended to evaluate tumor extent and regional spread. Additionally, further diagnostic tests, such as abdominal ultrasound and chest X-ray, as well as more recent methods like chest and abdominal CT scans, and PET-CT in selected cases, are commonly employed to assess for distant metastasis (5).

Historically, the main treatment for sinonasal malignancies consisted of external approaches like craniofacial resection or lateral rhinotomy with medial maxillectomy, possibly combined with adjuvant radiotherapy. However, over the past two decades, endoscopic endonasal surgery has emerged as the predominant approach, propelled by enhanced surgeon expertise, refined surgical techniques, and technological innovations such as high-definition endoscopes and image-guided procedures, thereby pushing the boundaries of what is achievable (6–9). Several studies have demonstrated that extended endoscopic surgery for ITAC is oncologically safe for most patients up to selected T4b cases, with limited morbidity (10, 11). Moreover, extensive evidence has consistently shown the superiority of this approach compared to external techniques in terms of margin status, local recurrence, and disease-specific survival (12–14). After endoscopic surgical treatment, adjuvant radiotherapy is frequently administered. Advances in radiotherapy, shifting from 3D conformal radiotherapy to intensity-modulated radiotherapy (IMRT), have minimized damage to surrounding healthy tissues (15).

The prognosis for sinonasal ITAC is relatively favorable, with reported 5-year overall survival (OS) and disease-specific survival (DSS) rates of 72.7 (67.3%-78.5%) and 80.0% (75.1%-85.5%) respectively (16). Nonetheless, recurrence rates of sinonasal ITAC remain as high as 50% in some cohorts (16).

The current study updates our previous experience (17, 18), and involves a substantial cohort with prolonged follow-up to further substantiate the efficacy of a treatment protocol consisting of endoscopic surgery followed by radiotherapy.

## 2 Materials and methods

### 2.1 Patient selection

This study spans thirty-one years from 1992 to 2022, during which 200 individuals with sinonasal ITAC were referred to our Department of Otorhinolaryngology, Head and Neck Surgery. All these patients underwent a state-of-the-art endoscopic resection in an academic tertiary reference center setting, followed by radiotherapy (RT) in the majority of cases. Those who received a different primary treatment, such as craniofacial resection (CFR) or lateral rhinotomy, or were deemed inoperable were excluded from the analysis. Among the 200 patients, 171 (85.5%) received their primary treatment at our department, while the remaining 29 patients underwent incomplete resection at a regional hospital before being referred to our institution for revision endoscopic surgery, conducted by one of our three senior anterior skull base surgeons (L.V.G., M.J. and V.V.P.). Data on a subset of 44 patients and later 123 patients were previously reported by Van Gerven et al. and Camp et al. (17, 18). This current cohort of 200 patients further updates the most recently reported experience on 123 patients (18).

Patient selection for endoscopic resection was based on a shared decision by the multidisciplinary Head and Neck tumor board and the patient, considering tumor extent, patient comorbidity, reconstructive requirements, and anticipated surgical morbidity and mortality. In summary, endoscopic resection was considered for all patients lacking dural, brain, or orbital invasion on preoperative imaging, consisting of CT and MRI. Exceptions included 9 T4b lesions exhibiting limited dural invasion, 2 of which showed minimal intracranial invasion, that were treated endoscopically.

### 2.2 Preoperative assessment

Diagnostic workup included histopathological examination of endoscopic biopsy specimens, with postoperative confirmation. Tumor extent was assessed preoperatively using contrast-enhanced CT primarily for bone evaluation and multiplanar MRI with gadolinium for further soft tissue evaluation. Tumors were staged according to the 8^th^ edition of the International Union for Cancer Control TNM classification of 2017 (19). Evaluation for regional and distant metastasis involved routine blood tests, liver function tests, in the early period chest X-rays and abdominal ultrasound scans, and more recently CT scans of the chest and abdomen, as well as FDG-positron emission tomography (PET) scans in selected patients.

### 2.3 Surgical approach

Endoscopic resection for T1 tumors involved *en bloc* removal following optimal exposure and identification of the tumor’s origin. For middle-sized tumors (T2), a phased resection approach was employed. Initially, the tumor bulk was visualized, and its origin was pinpointed using atraumatic suction during a preparatory phase. Subsequently, the bulk of the tumor was excised using either cold instruments or a microdebrider. Following tumor bulk reduction the identified tumor origin was broadly circumscribed and where possible and in the absence of critical anatomical structures a 1 cm mucosal margin was aimed at.

For larger tumors (T3–T4), a more extended “tumor disassembly” and subperiosteal centripetal resection implied a fronto-sphenoethmoidectomy and a resection of the superior segment of the nasal septum. In cases of unilateral tumors, tumors with a unilateral origin with a volume confined to one nasal cavity, contralateral radical surgery was not undertaken. Control biopsies at the cribriform plate were routinely conducted at the end of each surgery. In tumors abutting to but not clearly invading the cribriform plate and olfactory groove, the macroscopically uninvolved olfactory mucosa and olfactory fibers at the cribriform plate were routinely sampled. On rare occasions, this implied a limited CSF leakage that spontaneously stopped or was covered with fibrin sealant and fascia lata. Endoscopic reconstruction at the level of the ethmoid roof using a multilayer closure technique involving fascia lata and mucosa was exclusively performed in cases where limited dural invasion required dural resection.

Given the estimated regional metastasis prevalence at presentation of 1% or even lower, and the absence of evidence in the literature supporting prophylactic treatment of first-echelon lymph nodes (5), elective neck treatment was not performed.

### 2.4 Postoperative management

Adjuvant RT was preferred in most cases, except for select small T1 tumors with negative resection margins and patients with numerous comorbidities or advanced age. Intensity-modulated RT has been the standard since 2003, offering improved tissue sparing over conventional RT (15). For patients radiated postoperatively at our center, the prescribed total dose was 60 Gy in 30 daily fractions of 2 Gy (5 fractions per week). An additional boost volume up to 66-70 Gy was delineated in case of incomplete surgical resection (either microscopic or macroscopic). No elective irradiation of the cervical lymph nodes was performed (20). One patient was diagnosed with positive bilateral cervical lymph nodes, including a retropharyngeal adenopathy (N2c), on pre-radiation CT. This patient subsequently received adjuvant chemoradiotherapy.

### 2.5 Follow-up

Follow-up intervals were scheduled every two months during the first two years, every three months in the third year, and every four months in the fourth year, transitioning to bi-annual evaluations during the fifth year and annually thereafter. Each follow-up included a comprehensive clinical and endoscopic assessment of the nasal cavity, supplemented by imaging at predefined intervals. MRI of the sinonasal region was conducted every six months during the first five years. Whole-body imaging, such as PET-CT, was reserved for specific clinical indications or performed prior to initiating systemic therapy.

### 2.6 Statistical analysis

In this study, comprehensive clinical data, preoperative imaging records, surgical and histopathological reports, details regarding adjuvant therapy, and follow-up information were extracted from the electronic medical file. The Statistical Package for Social Sciences (SPSS, version 29.0.1.0, Chicago, IL) was utilized to process the collected data. Survival analyses were conducted to evaluate overall survival (OS), defined as the time to death from any cause; disease-specific survival (DSS), defined as the time to death specifically from ITAC; and local recurrence-free survival (LRFS), defined as the time to local tumor recurrence or death from any cause, using the Kaplan–Meier method. The time to each event was calculated from the date of primary surgery. Group comparisons in univariate analyses were performed using the Log-rank test, with statistical significance defined as a p-value of ≤.05. Cox proportional hazard models were applied to estimate the hazard ratios (HR) for individual variables, reported along with their corresponding 95% confidence intervals (CI). A forward selection procedure was employed to construct a multivariable model of independent predictors, requiring a significance level of 5% for variables to be included in the model.

## 3 Results

A total of 200 patients were included in the study. The age at diagnosis ranged from 39 to 93 years, mean 64.3, median 64 years. Patients were followed for a mean and median of 93.2 and 78 months respectively (range = 1–336 months). The mean and median follow-up time for local recurrence-free patients was 80.7 and 64 months respectively (range = 1–329 months). Wood dust exposure emerged as the primary etiological factor for ITAC, with only eight patients (4%) claiming not to have been exposed to wood dust in our cohort. The occupational hazard led to a significant disparity in the gender distribution, with a male-to-female ratio of 197:3. The olfactory cleft was the most commonly affected site, with 81.5% of tumors originating in this region. Cribriform plate involvement was reported in 68 patients.

The TNM classification at initial diagnosis is summarized in **Table 1**. None of the patients presented with distant metastatic disease at first presentation. However, one patient, with a T4a primary tumor, had regional metastatic disease, involving the contralateral neck, classified as N2c. During follow-up, distant metastases developed in 20 cases (10.0%), occurring at multiple sites (n=7), or isolated in the lung (n=5), brain (n=4), bone (n=2), liver (n=1), or spinal cord (n=1). The mean and median time between first treatment and development of distant metastasis was 34.6 and 20.5 months respectively (range 1-114 months). Only two patients (1.0%) developed cervical lymph node metastasis during follow-up.

**Table 1:** Patient, tumor, and treatment characteristics.

Total cohort		N	%
		200	100
Patient characteristics			
Age at diagnosis (years)	<55	38	19.0
	55-64	64	32.0
	65-74	63	31.5
	>75	35	17.5
Sex (male)		197	98.5
Wood dust exposure	Yes	183	91.5
	No	8	4.0
	Undefined	9	4.5
Tumor characteristics			
T-classification	T1	29	14.5
	T2	85	42.5
	T3	42	21.0
	T4	44	22.0
	T4a	35	17.5
	T4b	9	4.5
N-classification		1 (N2c)	0.5
M-classification		0	0
Tumor differentiation	Well-differentiated	40	20.0
	Moderately differentiated	42	21.0
	Poorly differentiated	39	19.5
	Signet cell differentiation	15	7.5
	Undefined	79	39.5
Lamina cribrosa involvement	Yes	68	34.0
	No	122	61.0
	Undefined	10	5.0
Treatment characteristics			
Positive margins		24	12.0
Adjuvant radiotherapy		187	93.5

**Figure 1:**
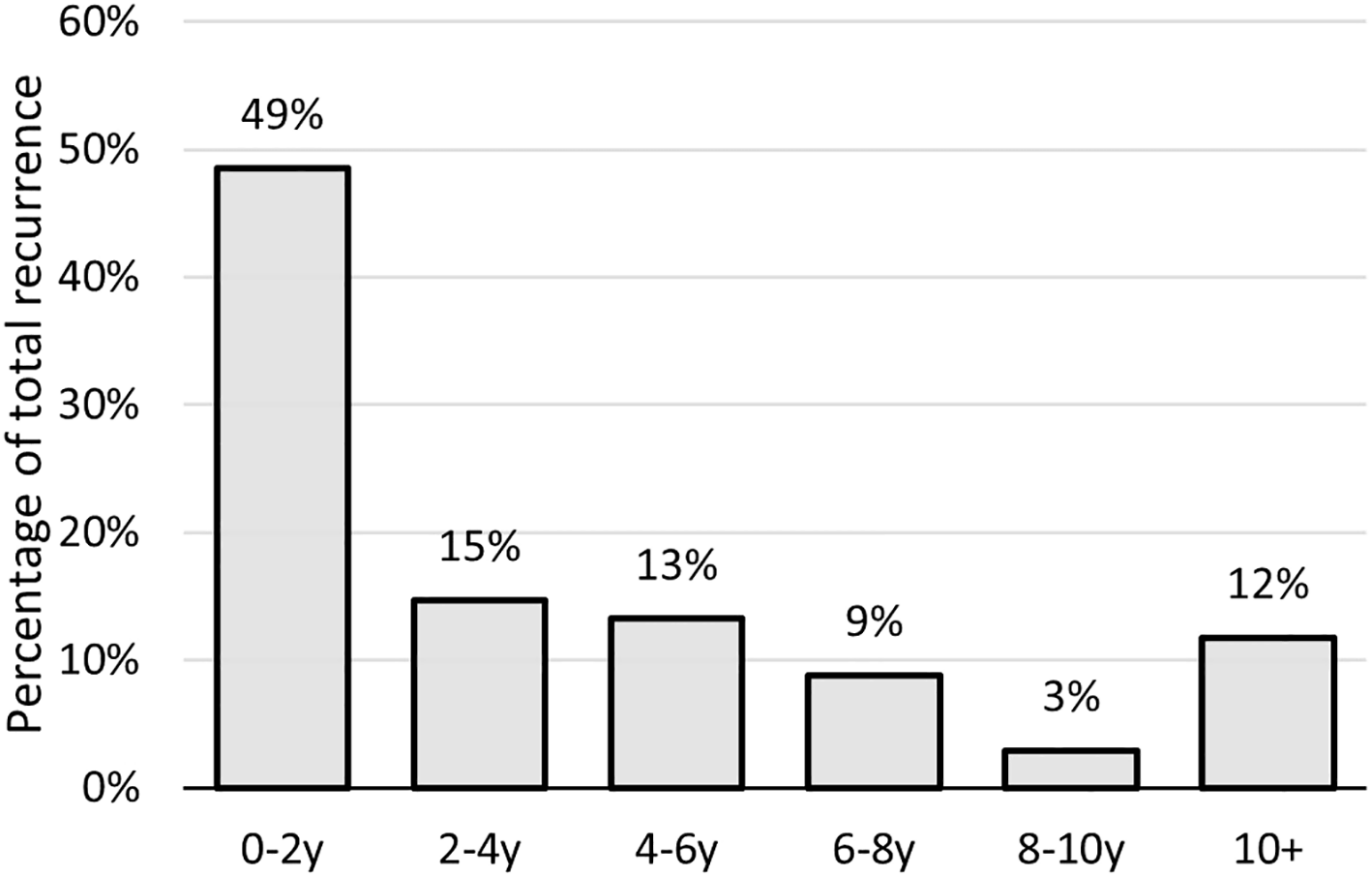
Time to recurrence/second primary of sinonasal ITAC in years after treatment; ITAC, intestinal-type adenocarcinoma.

Local recurrence occurred in 68 patients (34.0%), predominantly in the posterior ethmoid (46.2%), fovea ethmoidalis (33.8%), and posterior septum (32.3%). Unlike the primary tumor, the first local recurrence less frequently originated in the olfactory cleft and relatively more in the sphenoid sinus (13.6% vs 3.0% for the primary tumor). Thirty-two patients developed a second recurrence, and seventeen had a third recurrence. The mean time to first local recurrence was 50.7 months, while the median time interval was 26 months (range 5-247 months) (**Figure 1**). Therapy of the first local recurrence was predominantly surgery (91.2%), followed by radiotherapy in cases with no previous radiation, and by chemotherapy in 3 other patients. A combined endoscopic and external approach resection was performed in six of these patients (8.8%). Chemotherapy alone was administered in another three patients. Two patients underwent debulking surgeries to alleviate discomfort caused by recurrent tumors that were deemed inoperable.

At the last follow-up, 47.5% of patients were alive, 20.5% had succumbed to the disease and 32.0% to intercurrent illnesses. The 5-year and 10-year overall survival (OS), disease-specific survival (DSS), and local recurrence-free survival (LRFS) rates were analyzed, with the respective rates reported as follows: 5-year OS: 71.4% (± 3.4%), 10-year OS: 48.2% (± 4.1%); 5-year DSS 85.1% (± 2.8%), 10-year DSS: 76.2% (± 3.8%); 5-year LRFS: 55.2% (± 3.7%), 10-year LRFS: 32.2% (± 3.8%).

**Figure 2:**
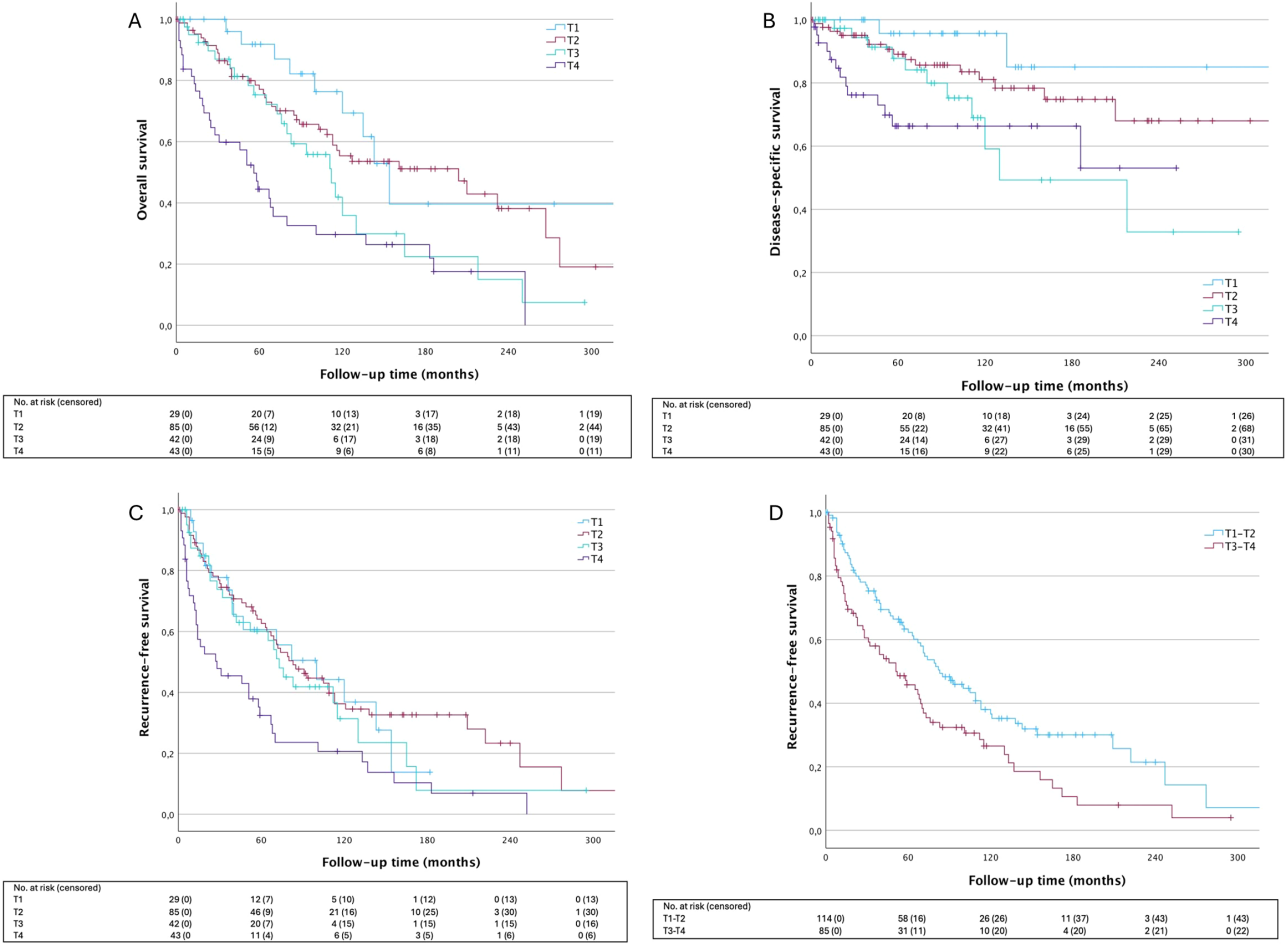
Survival outcomes according to T-lassification. **(A)** OS HR_T2_ 1.53 [95% CI: 0.74-3.17], p=.249; HR_T3_ 2.34 [95% CI: 1.08-5.09], p=.32; HR_T4_ 3.96 [95% CI:1.87-8.37], p=.0003; **(B)** DSS HR_T2_ 2.459 [95% CI: 0.56-10.77], p=.2324; HR_T3_ 4.63 [95% CI: 1.02-20.90], p=.0465; HR_T4_ 6.24 [95% CI: 1.41-27.69], p=.0161; **(C)** LRFS OS HR_T2_ 0.94 [95% CI: 0.53-1.64], p=.8185; HR_T3_ 1.12 [95% CI: 0.60- 2.10], p=.7132; HR_T4_ 2.06 [95% CI: 1.14-3.71], p=.0163; **(D)** LRFS HR_T3-4_ 1.61 [95% CI: 1.15-2.27], p=.0061. T1 is used as reference for analysis in **(A–C)**, T1-2 is used as reference in **(D)**. OS, overall survival; DDS, disease-specific survival; LRFS, local recurrence-free survival.

In univariate analysis, significant differences in OS and DSS were observed among different T-classifications at presentation (**Figure 2**). Specifically, T1 tumors demonstrated significantly better OS compared to T3 (HR: 2.34 [95% CI: 1.08–5.09], p=.032) and T4 tumors (HR: 3.96 [95% CI: 1.87–8.37], p=.0003), as well as improved DSS compared to T3 (HR: 4.63 [95% CI: 1.02–20.90], p=.047) and T4 tumors (HR: 6.27 [95% CI: 1.41–27.69], p=.016). In terms of LRFS, T1, T2, and T3 tumors showed a more favorable prognosis compared to T4 tumors (p=.027, p<.001, and p=.028, respectively). When categorizing tumors into small (T1–T2) and large (T3–T4) groups, T-classification had a pronounced significant effect on OS (HR: 2.19 [95% CI: 1.48–3.24], p<.0001), DSS (HR: 2.57 [95% CI: 1.37–4.79], p=.0031), and LRFS (HR: 1.61 [95% CI: 1.15–2.27], p=.0061).

Local recurrence had a significant negative impact on OS (HR: 1.96 [95% CI: 1.27–3.02], p=.0023), and DSS (HR: 12.28 [95% CI: 5.59–26.99], p<.0001). An even stronger association was observed between the development of distant metastasis and OS (HR: 11.96 [95% CI: 7.23–19.76], p<.0001) and DSS (HR: 41.17 [95% CI: 21.58–78.55], p<.0001).

**Figure 3:**
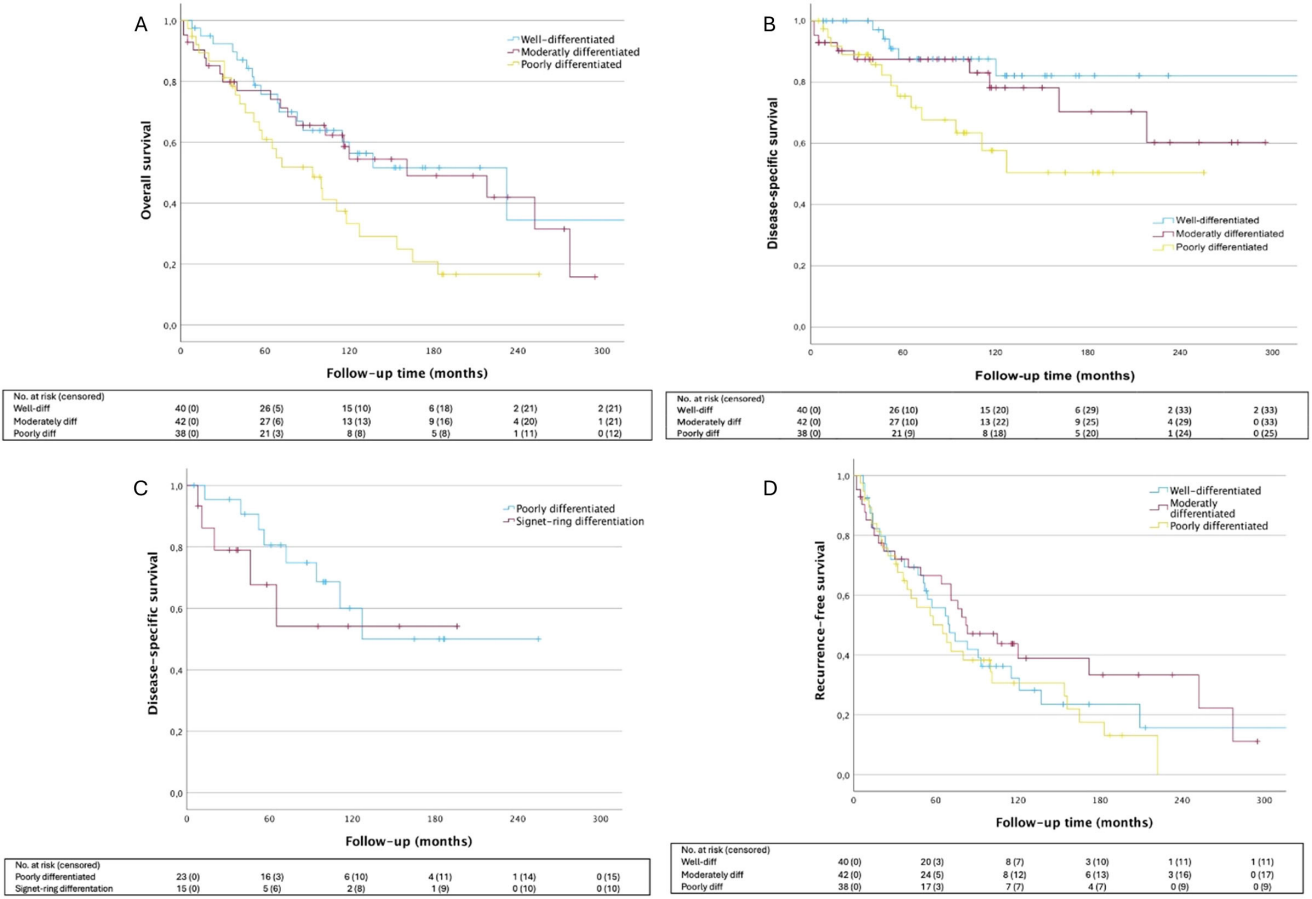
Survival outcomes according to tumor differentiation. **(A)** OS HR_mod_ 1.16 [95% CI: 0.61-2.22], p=.6497; HR_poor_ 2.08 [95% CI: 1.12-3.84], p=.0202; **(B)** DSS HR_mod_ 1.80 [95% CI: 0.60-5.39], p=.2940; HR_poor_ 3.38 [95%CI: 1.20-9.51, p=.0209; **(C)** DSS HR_poor_ 2.96 [95% CI: 0.97-9.05], p=.0575; HR_sign_ 4.42[95% CI: 1.27-15.39], p=.0197; **(D)** LRFS HR_mod_ 0.78 [95% CI: 0.46-1.35], p=.3802; HR_poor_ 1.18 [95% CI: 0.70-1.99], p=.5351. Well-differentiated tumor group is used as the reference for all analysis. OS, overall survival; DSS, disease-specific survival; LRFS, local recurrence-free survival.

Well-differentiated tumors exhibited significantly better OS and DSS compared to poorly differentiated tumors (HR: 2.08 [95% CI: 1.12–3.84], p=.0202 and HR: 3.38 [95% CI: 1.20–9.51], p=.0209, respectively) (**Figure 3**). Similarly, moderately differentiated tumors had improved OS compared to poorly differentiated ones (p=.048). When analyzing signet-ring differentiation independently, we observed a trend toward worse DSS compared to other poorly differentiated tumors with respective 5-year DSS rates of 67.7% (± 14.0%) and 80.6% (± 8.7%). Nonetheless, this difference did not achieve significance overall (p=.456). No significant differences were seen in the local recurrence rate in relation to the histological grade of differentiation (p=.330), however, distant metastasis occurred more frequently in poorly differentiated tumors (25.6%) compared to moderately differentiated (9.5%) and well-differentiated tumors (2.5%) (p=.006).

Moreover, the status of the cribriform plate was investigated regarding resection margins. Tumor involvement at this site was associated with reduced OS (HR: 2.24 [95% CI: 1.49–3.38], p=.0001), DSS (HR: 3.04 [95% CI: 1.60–5.78], p=.0007), and LFRS (HR: 1.752 [95% CI: 1.22–2.51], p=.0023), and a greater need for salvage surgical procedures.

In terms of treatment-related factors, we found that patients who underwent a non-radical resection followed by radiotherapy had lower OS (HR: 1.89 [95% CI: 1.00–3.58], p=.0491), DSS (HR: 2.95 [95% CI: 1.29–6.74], p=.0106), and LRFS (HR: 2.15 [95% CI: 1.26–3.67], p=.0051) compared to patients with negative section margins and adjuvant radiotherapy. Distant metastasis was also more frequent in this group (p=.019). Conversely, our survival analysis could not demonstrate a significant difference regarding OS, DSS, or LRFS between patients who previously underwent an incomplete resection in a regional hospital before being referred to our department for completion of endoscopic surgery.

OS is described by a multivariable model in which independent predictors include age at diagnosis, T-status, and tumor differentiation (**Table 2**). For DSS, after adjusting for other variables, independent prognostic factors include pooled tumor size (small, T1-T2, versus large tumors, T3-T4) and positive surgical margins. Age at diagnosis, T-status, and positive surgical margins were identified as independent predictors for LRFS.

**Table 2:** Multivariable model of baseline characteristics.

Variable	Category	HR (95% CI)	P-value
OS			
Age at diagnosis (+1 year)		**1.071 (1.048–1.094)**	**<.0001**
Tumor size (T-status)	T1	1	
	T2	1.900 (0.908–3.977)	.0886
	T3	**2.630 (1.199–5.766)**	**.0158**
	T4	**4.573 (2.134–9.799)**	**<.0001**
Tumor differentiation	Well-differentiated	1	
	Moderately differentiated	1.790 (0.919–3.485)	.0869
	Poorly differentiated	**2.428 (1.304–4.519)**	**.0052**
DSS			
Tumor size pooled	T1-2	1	
	T3-4	**2.377 (1.263–4.473)**	**.0073**
Positive surgical margins	No	1	
	Yes	**2.436 (1.056–5.624)**	**.0369**
LRFS			
Age at diagnosis (+1 year)		**1.049 (1.031–1.068)**	**<.0001**
Tumor size (T-status)	T1	1	
	T2	1.127 (0.640–1.987)	.6784
	T3	1.155 (0.617–2.164)	.6526
	T4	**2.357 (1.286–4.322)**	**.0056**
Positive surgical margins	No	1	
	Yes	**1.777 (1.019–3.100)**	**.0428**

A 5% significance level was adopted for variables to enter the model. OS, Overall survival; DSS, Disease-specific-survival; LRFS, Local recurrence-free survival; HR, Hazard ratio; CI, Confidence interval. Significant findings are indicated in bold.

The majority of patients (93.5%) received postoperative RT (60 or 66Gy in most cases). One patient received pre-operative RT at a regional hospital before referral and two patients were treated with adjuvant concomitant chemoradiotherapy, one because of bilateral neck involvement (N2c) and one because of synchronous esophageal cancer. When examining the patients who did not receive radiotherapy, we noticed an increased local recurrence rate of 38.5% compared to 34.1% in the surgery plus radiotherapy group (p=.059), despite a selection bias of more extended primary tumors being selected for postoperative RT.

## 4 Discussion

Previous research demonstrated favorable 5-year DSS and LRFS for endoscopically treated ITAC (11, 13, 21–23). Our findings, indicating a 5-year DSS of 85.1% and a 5-year LRFS of 55.2%, align closely with these established reports. Whereas previously we could not find a statistically significant influence of T-classification (18), the present study, through multivariable analysis, establishes T-classification as an independent prognostic factor for OS, DSS, and LRFS.

The literature has identified pathological prognostic factors. The presence of signet-ring cells has been shown to correlate with unfavorable prognosis and overall poorly differentiated tumors are associated with shorter recurrence-free survival compared to well- and moderately differentiated tumors and are prone to distant relapse (13, 21, 24–26). Indeed, our results confirm that poorly differentiated tumors have a trend toward a reduced OS and DSS compared to well-differentiated tumors. After adjusting for other variables, tumor differentiation remained a significant predictor of OS. Although not reaching statistical significance, the signet-ring cell subtype appeared to be associated with the poorest DSS among poorly differentiated tumors. Distant metastasis occurred most frequently in poorly differentiated tumors. Among the 15 patients with metastatic disease and documented differentiation status, 10 were poorly differentiated, 4 were moderately differentiated, and 1 was well-differentiated. Conversely, contrary to what has been reported prior to this study (21), no difference in local recurrence was observed between histological subgroups in our cohort.

Lastly, we examined treatment-related factors. Positive margins have been demonstrated to be a poor prognostic factor for local recurrence (11, 13). This finding was confirmed in our study with 5-year LRFS dropping from 89.0% (± 2.6%) to 61.2% (± 14.8%) in case of positive tumor margin status. For DSS and LRFS, incomplete resection before adjuvant radiotherapy was independently associated with a worse prognosis. Cribriform plate involvement, a common contributor to positive margins, is a well-established prognostic factor in sinonasal cancer. Numerous studies have demonstrated its association with reduced survival and higher recurrence rates (18, 23, 27). This is likely due to the inherent difficulties of achieving clear resection margins in this anatomically complex region, which significantly elevates the risk of local recurrence, a finding corroborated by the results of our study. Studies published over the last two decades have demonstrated the feasibility and efficacy of more radical strategies, such as the endoscopic endonasal transcribriform approach (EETA), for achieving clear margins in locally advanced sinonasal malignancies. EETA extends surgery to the anterior skull base, enabling precise en bloc resections and reducing the risk of microscopic residual disease (10, 28, 29). Although the detailed evaluation of EETA is beyond the scope of this paper, our findings underline the pressing need for innovative approaches to improve the radicality of surgery in patients with cribriform plate involvement. Ensuring negative margins before adjuvant therapy is crucial and therefore treatment of ITAC should be reserved for centers of excellence capable of performing extensive resections when needed.

We analyzed data from 29 patients who initially underwent incomplete resection at a regional hospital before being referred to our center for completion surgery. In these patients, unilateral polyps were identified as benign pathology, and conventional endoscopic surgery was performed. Following the pathology reports indicating malignancy, patients were referred for further treatment. This subgroup did not exhibit significantly inferior outcomes in terms of OS and DSS compared to the 171 patients initially treated at our tertiary center, and contrary to what we demonstrated in our previous research LRFS is also not affected. Thus, we were unable to demonstrate the negative impact of possible transtumoral dissection and tumor seeding in the sinonasal tract. This association remained non-significant even after adjustment in the multivariable analysis. Possible explanations for this could be the lack of statistical power in our analysis due to a small number of patients in the referral group.

The role of adjuvant radiotherapy for ITAC has been a topic of discussion. The existing evidence advocates for adjuvant radiotherapy in large tumors (T3-T4), positive margins, and poor histological differentiation (17). At our center, adjuvant radiotherapy was generally recommended for most patients by the multidisciplinary Head and Neck tumor board. Only 13 patients did not undergo radiation, mostly because of advanced age or co-morbidities rather than T- or margin status. Consequently, no pertinent conclusions could be drawn as to the contribution of postoperative radiotherapy to survival. Nonetheless, a trend toward increased local recurrence was observed in the non-radiation group, without impacting overall or disease-specific survival. Further research is warranted to elucidate this matter and derive relevant conclusions.

We found great heterogeneity in the time of local recurrence in our cohort. Although roughly half of the recurrences occurred within the first 2 years after treatment, more than 10% were diagnosed more than a decade after the initial follow-up period. This finding underscores that late recurrences or second primaries are not uncommon, highlighting the need for extended and vigilant long-term monitoring of patients. In our study population, local recurrence significantly affected overall survival.

The tendency of metastatic expansion is well documented in ITAC. Our data reveals that 10.0% of patients develop metastasis during the follow-up period, with a median duration from initial diagnosis to detection of distant disease being only 20.5 months. This finding could argue for more surveillance during the follow-up period, perhaps tailored to the differentiation status of the primary tumor. Moreover, based on our data, additional risk factors for metastasis include advanced-stage tumors and positive margins. In this context, it might be advisable to implement a customized surveillance plan that is adjusted to the specific characteristics of the tumor and treatment, rather than adopting a one-size-fits-all approach. The impact of this on survival may be limited, however.

The study is subject to several limitations, including the retrospective collection of data, the exclusion of inoperable patients, the prolonged duration of inclusion, and the potential classification bias due to the extended follow-up period.

## 5 Conclusion

In conclusion, our study focused exclusively on patients diagnosed with ITAC who underwent endoscopic treatment followed by radiotherapy. Our findings align closely with previous reports regarding favorable 5-year OS, DSS, and LRFS for endoscopically treated ITAC. Notably, we observed reduced OS and DSS for poorly differentiated tumors, with the signet-ring cell subtype showing a trend toward the poorest DSS. Moreover, poorly differentiated tumors exhibited a higher incidence of metastatic disease. Positive margins are a poor prognostic factor for local recurrence and a risk factor for distant metastasis. Ensuring negative margins should set the benchmark for surgical best practices. Nearly half of all local recurrences occur within the first two years of follow-up, but since over 10% occur after 10 years, lifelong follow-up is warranted.

## Data Availability

The raw data supporting the conclusions of this article will be made available by the authors, without undue reservation.
